# CYP2E1 in Alcoholic and Non-Alcoholic Liver Injury. Roles of ROS, Reactive Intermediates and Lipid Overload

**DOI:** 10.3390/ijms22158221

**Published:** 2021-07-30

**Authors:** Riina Harjumäki, Chris S. Pridgeon, Magnus Ingelman-Sundberg

**Affiliations:** 1 Section of Pharmacogenetics, Department of Physiology and Pharmacology, Karolinska Institutet, 171 65 Stockholm, Sweden; riina.harjumaki@ki.se (R.H.); christopher.pridgeon@ki.se (C.S.P.); 2 Biopharmaceutics Group, Department of Pharmaceutical Biosciences, Faculty of Pharmacy, University of Helsinki, 07900 Helsinki, Finland

**Keywords:** NASH, NAFLD, liver, ROS, NAFL, lipid peroxidation

## Abstract

CYP2E1 is one of the fifty-seven cytochrome P450 genes in the human genome and is highly conserved. CYP2E1 is a unique P450 enzyme because its heme iron is constitutively in the high spin state, allowing direct reduction of, e.g., dioxygen, causing the formation of a variety of reactive oxygen species and reduction of xenobiotics to toxic products. The CYP2E1 enzyme has been the focus of scientific interest due to (i) its important endogenous function in liver homeostasis, (ii) its ability to activate procarcinogens and to convert certain drugs, e.g., paracetamol and anesthetics, to cytotoxic end products, (iii) its unique ability to effectively reduce dioxygen to radical species causing liver injury, (iv) its capability to reduce compounds, often generating radical intermediates of direct toxic or indirect immunotoxic properties and (v) its contribution to the development of alcoholic liver disease, steatosis and NASH. In this overview, we present the discovery of the enzyme and studies in humans, 3D liver systems and genetically modified mice to disclose its function and clinical relevance. Induction of the CYP2E1 enzyme either by alcohol or high-fat diet leads to increased severity of liver pathology and likelihood to develop ALD and NASH, with subsequent influence on the occurrence of hepatocellular cancer. Thus, fat-dependent induction of the enzyme might provide a link between steatosis and fibrosis in the liver. We conclude that CYP2E1 has many important physiological functions and is a key enzyme for hepatic carcinogenesis, drug toxicity and liver disease.

## 1. The Discovery of CYP2E1

Early studies of the metabolism of ethanol, independent of alcohol dehydrogenase (ADH), were performed using a mutant strain of deermouse (*Peromyscus maniculatus*) that is genetically deficient in low-Km ADH. Nonetheless, these mice were found to eliminate ethanol by a previously unidentified enzyme system. One of the enzyme candidates proposed was a cytochrome P450 active in ethanol oxidation in liver microsomes [1], which was also metabolized other short chain alcohols [2] and appeared to be inducible by ethanol treatment in animal models [3].

The exact mechanisms of P450-dependent ethanol oxidation were initially unclear but were associated with the production of reactive oxidative species (ROS), including hydroxyl radicals, which can indirectly oxidize ethanol [4,5]. The finding of an ethanol-dependent increase in P450-mediated oxidation [3,6] was important for the isolation of a specific form of ethanol inducible P450 and to understand the direct P450-mediated oxidation of ethanol. One such enzyme was purified from ethanol- and benzene-induced rabbits and from ethanol-treated rats [7,8]. This enzyme had the highest ethanol oxidation capacity of several different P450 forms isolated [7]. The corresponding cDNA was cloned in 1987 [9] and the enzyme was named cytochrome P450 2E1 (CYP2E1) in 1991 [10].

## 2. Expression, Functions and Cellular Fate of CYP2E1

CYP2E1 is well conserved across mammalian species, indicating an important physiological function. In humans, the *CYP2E1* gene has nine exons located on chromosome 10 and spans 11,413 base pairs [11]. Substantial inter-ethnic polymorphisms exist in *CYP2E1*. However, only very rare variants causing amino acid shifts have been described [12], and despite several epidemiological studies there is no clear evidence that any polymorphic variants have any functional relevance [13,14]. One recent study suggested a link between *CYP2E1*-333A > T and NASH, the authors showed increased inflammation and NASH in patient biopsies with the TA allele, largely mediated by a small increase in interferon-inducible protein 10 [15]. However, because only a relatively low number of patients were studied, much credence cannot be given to these findings unless they are reproduced independently. In liver, CYP2E1 is expressed mainly in the endoplasmic reticulum (ER) of the hepatocytes, but is also found in the hepatic Kupffer cells [16,17,18]. Hepatic CYP2E1 is also present in the mitochondria, by translocation, following expression in the nuclear genome [19,20,21] and the plasma membrane [19,22,23,24]. However, the magnitude of induction of CYP2E1 in mitochondria is smaller than that of ER-resident CYP2E1.

CYP2E1 metabolizes a variety of small, hydrophobic substrates and drugs (reviewed in [13,14,21,25]). It is responsible for the metabolism of many toxic or carcinogenic chemicals, including chloroform and benzene, and drugs, such as paracetamol, salicylic acid and several inhalational anesthetics (e.g., isoflurane, sevoflurane and halothane). It therefore follows that conditions which elevate the expression of CYP2E1 can increase the damage caused by conversion of drugs to toxic intermediates [26]. The bioactivation of several pre-carcinogens by CYP2E1 has been discussed in relation to the development of cancers, particularly hepatocellular carcinoma (HCC) [27,28]. In addition, CYP2E1 metabolizes endogenous substances including acetone, acetol, steroids and polyunsaturated fatty acids, such as linoleic acid and arachidonic acid to generate ω-hydroxylated fatty acids [29,30,31,32]. CYP2E1 also metabolizes ethanol and other short chain alcohols, however its contribution to the overall ethanol clearance is low and P450-dependent alcohol metabolism does not influence overall ethanol clearance in rats [33]. Despite its importance as a metabolic enzyme the crystal structure and binding sites were only determined relatively recently, in 2008 [34,35].

**Figure 1:**
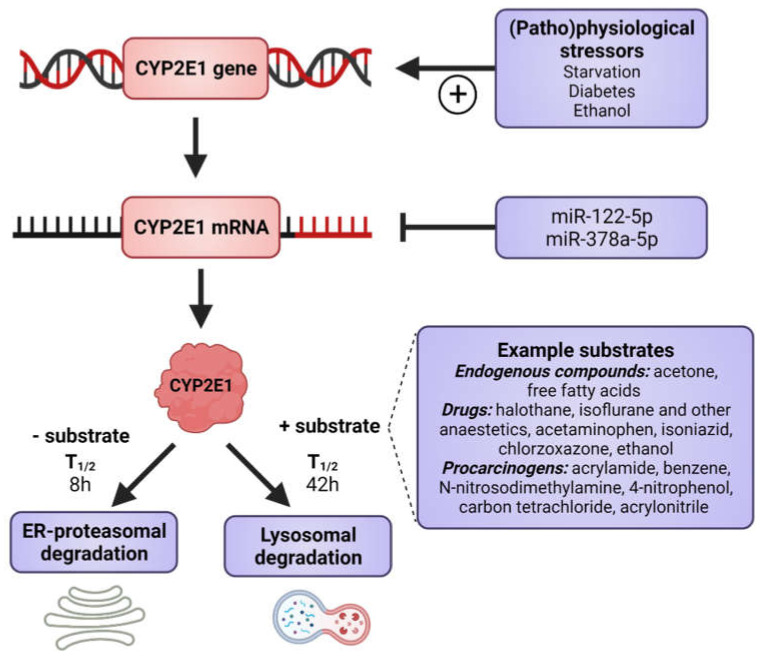
Factors that induce CYP2E1 expressions. CYP2E1 can be induced by multiple mechanisms in transcriptional, post-transcriptional, translational, and post-translational levels. Some physiological and pathophysiological conditions, such as starvation and uncontrolled diabetes, increase CYP2E1 on the transcriptional level. Many hormones and cytokines regulate CYP2E1 on the mRNA and protein expression levels. Enzyme substrates stabilize the enzyme, preventing degradation by the ER proteasome and the enzyme degradation instead takes place via the autophagosome-lysosomal pathway. Figure made using BioRender.

CYP2E1 is regulated by multiple, distinct mechanisms at the transcriptional, post-transcriptional, translational, and post-translational levels (Figure 1) [36,37,38,39,40]. CYP2E1 expression is elevated in response to a variety of physiological and pathophysiological conditions, such as starvation and uncontrolled diabetes, and also by ethanol, acetone and several other low molecular weight substrates [7,29,41,42,43,44,45]. However, it is primarily regulated at the post-transcriptional and post-translational levels [46]. Substrate-induced enzyme stabilization is the most important regulatory mechanism for CYP2E1 [47,48]; substrates and other chemicals binding to the substrate binding region stabilize the enzyme and prevent degradation by the proteasome-ER complex [38,48,49], involving the UBC7/gp78 and UbcH5a/CHIP E2-E3 ubiquitin ligases [50]. Thus, ER-mediated degradation is mainly active on CYP2E1 in the absence of substrates, whereas ligand-stabilized CYP2E1 is degraded more slowly by the autophagic-lysosomal pathway [51]. Due to its important physiological functions, the expression of CYP2E1 is under tight homeostatic control and multiple endogenous factors regulate CYP2E1 mRNA stability and protein expression including hormones (such as insulin, glucagon, growth hormone, adiponectin and leptin), growth factors (such as epidermal growth factor and hepatocyte growth factor) and various cytokines (reviewed in [40]).

As previously mentioned, the *CYP2E1* gene is highly conserved and no functionally important genetic variants have been described. This indicates an important endogenous role, which is supported by our findings, wherein the knockdown of human *CYP2E1* expression in the in vivo relevant three-dimensional (3D) liver spheroids [52] causes dramatic cell death in hepatocytes (unpublished observations from our lab). It is clear that CYP2E1 has important effects during catabolic conditions as the gene is transcriptionally induced during starvation [41]. The hepatic production of glucose is essential under starvation conditions with the primary supply of brain glucose, and about 10% of plasma glucose originating from acetone [53]. CYP2E1 readily oxidizes acetone to acetol [36], which is subsequently converted to pyruvate and then to glucose during gluconeogenesis. In addition, during conditions of starvation energy supply from fatty acids is essential and CYP2E1 is efficient in the ω-oxidation of fatty acids.

## 3. Mechanisms of Action of CYP2E1; Radical Mediated Toxicity

CYP2E1 is unique in that its heme iron is constitutively in the high spin state. In the cytochrome P450 redox cycle, substrate binding is necessary in order to transfer the low spin form into a high spin form. The conversion of low spin (three electrons in the outer Fe^3+^ shell with opposite spin) to a high spin (parallel spin of the three outer shell electrons) form of P450 can be determined by absorption spectra analyses where the high spin form has a peak at 390 nm. The constitutive high spin form of CYP2E1 facilitates electron transfer to dioxygen in the absence of the substrate and is the major reason for CYP2E1 being a ‘leaky’ enzyme which generates ROS such as superoxide and hydrogen peroxide. In the presence of iron, hydrogen peroxide is split yielding hydroxyl radicals formed in an iron-catalyzed Haber-Weiss reaction. Such reactions occur spontaneously in an environment enriched in hydrogen peroxide and non-heme iron. The hydroxyl radicals generated via the action of CYP2E1 can react with the hydrogen on the α-carbon of ethanol yielding a cytotoxic ethanol radical [54], this radical is oxidized spontaneously to acetaldehyde.

**Figure 2:**
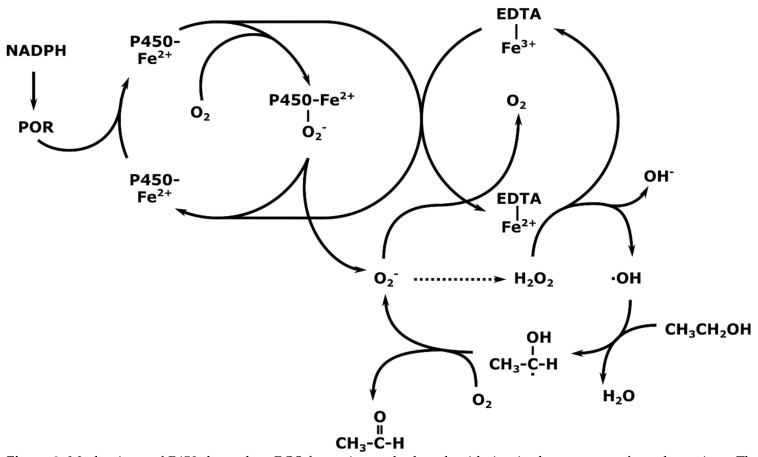
Mechanisms of P450-dependent ROS formation and ethanol oxidation in the presence of non-heme iron. The generated superoxide is dismutated to hydrogen peroxide (dashed arrow), which is cleaved in the presence of non-heme iron (Fe^2+^) yielding hydroxyl radicals. These can extract the hydrogen on the α-carbon of ethanol creating a radical of ethanol which is spontaneously converted to acetaldehyde. Non-heme iron chelated to EDTA enhances the cleavage of hydrogen peroxide yielding the reactive hydroxyl radical. Basic mechanism as described in [54]. POR-P450 reductase.

The extent of contribution of non-heme Fe^2+^ in the reaction cycle of CYP2E1 is unknown. The enzyme can oxidize several different substrates based on a conventional cytochrome P450 redox cycle and it is presumed that both reaction mechanisms work in parallel with regard to ethanol and other short chain aliphatic alcohols. Thus, it is difficult to distinguish ROS production by CYP2E1 from ROS production from the uncoupling of the nicotinamide adenine dinucleotide phosphate acid (NADPH)-dependent electron transport chain and cytochrome P450 reductase. Accordingly, many experiments have been conducted in the presence of EDTA-chelated iron, which causes uncoupling and nonspecific generation of ROS, including the hydroxyl radical by extracting a proton from ethanol, yielding acetaldehyde (Figure 2) [4,55]. As such, determination of the true contribution of CYP2E1-mediated ROS formation and effects on the subsequent development of alcoholic liver disease (ALD) has been based on the use of CYP2E1 specific antibodies, knockdowns and *CYP2E1* transgenic animals.

**Figure 3:**
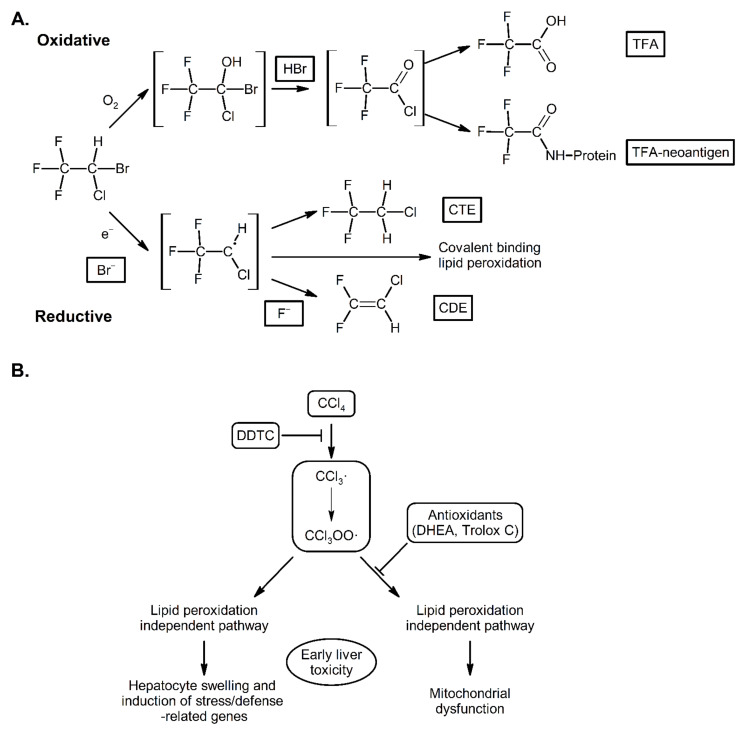
Mechanisms of CYP2E1 dependent oxidation and reduction of halothane [60,61] (**A**) and carbon tetrachloride [62] (**B**). Aerobically, halothane undergoes cytochrome P450 catalyzed oxidation to trifluoroacetic acid (TFA), bromide and a reactive intermediate that can acetylate liver proteins and produces neo-antigens which stimulate an immune reaction that mediates severe hepatic necrosis. Anaerobically, halothane is reduced to a radical that can generate the metabolites chlorotrifluoroethane (CTE) and chlorodifluoroethylene (CDE) and also covalently bind proteins generating autoimmune reactions and also induce lipid peroxidation.

The high spin nature of the CYP2E1 heme iron also makes the enzyme unique in that it can reduce compounds. The most well-known reaction is the reduction of carbon tetrachloride into the corresponding radicals that efficiently induce lipid peroxidation [56]. This reaction is believed to be the major cause of the hepatotoxicity of carbon tetrachloride. In addition, halothane is reduced in a similar manner by CYPE1 to a reactive radical and binding of this radical metabolite to CYP2E1 converts the enzyme into a cell surface autoantigen believed to contribute to halothane hepatitis (Figure 3) [29]. In addition to halothane, other anesthetics, such as enflurane, isoflurane and desflurane, are metabolized by CYP2E1 to trifluoroacetylated components some of which may be immunogenic [57,58]. Similar autoimmune consequences of CYP2E1 action are also found in CYP2E1-mediated formation of hydroxyethyl radicals which bind to cellular proteins causing the production of autoantibodies in alcoholics [59].

## 4. CYP2E1 in ALD

ALD is the most frequent liver disease in Europe, causing approximately 500,000 deaths per year, HCC resulting from pathological changes in the liver contributes highly to this death rate. There are strong genetic determinants in the development of ALD, e.g., *PNPLA3*, *TM6SF2*, and only approximately 10–20% of alcoholics develop cirrhosis of the liver [63].

ALD and NAFLD have several common mechanisms; generally, fibrosis occurs in response to enhanced ROS levels, lipid mediators and pro-inflammatory cytokines. Thus, ALD and NAFLD are mechanistically similar and share histopathological features, particularly in terms of CYP2E1 induction and oxidative stress (reviewed in [64]). In addition, they share common genetic risk factors.

CYP2E1 is the most relevant CYP in ALD [65], it is highly inducible, has high catalytic activity for ethanol [25] and is prone to futile cycling in the absence of substrate to produce ROS [66]. CYP2E1 is regarded as a ‘leaky’ enzyme due to loose coupling of the CYP redox cycle, permitted by the constitutively high-spin state of the heme iron and, therefore, it has a great capacity to produce oxyradicals and initiate lipid peroxidation [67,68,69,70,71]. Thus, CYP2E1 may be important in mediating the effects of ethanol on ALD via increased lipid peroxidation [67].

In order to study the link between CYP2E1 and ALD, Morgan et al., generated *CYP2E1* transgenic mice which exhibited increased serum ALT levels, higher histological scoring and ballooning hepatocytes with alcohol diet [72]. Using a transgenic mouse model expressing extra copies of human *CYP2E1*, Butura et al., showed increased liver injury and expression of stress related genes with alcohol diet [73]. Microarray analyses revealed that enhanced expression of structural genes, particularly cytokeratin 8 and 18, may be related to the observed pathology and they were suggested as biomarkers for ALD [73]. JunD, part of the transcription factor complex AP-1, was induced by CYP2E1 and alcohol, and its expression correlated with the degree of liver injury. This transcription factor complex is also linked to increased macrophage activation. Furthermore, JunD also has a role in hepatic stellate cell activation and regulates the cytokine interleukin 6 [73].

A second approach is to study *CYP2E1* knockout mice in comparison to wild-type mice. Abdelmegeed et al., showed that that aged wild-type mice had increased hepatocyte vacuolation, ballooning, degeneration, and inflammatory cell infiltration compared with *CYP2E1*-null mice [74]. They also found that the aged wild-type mice had increased hepatocyte apoptosis, hepatic fibrosis, levels of hepatic hydrogen peroxide, lipid peroxidation, protein carbonylation, nitration and oxidative DNA damage, indicating an endogenous role for CYP2E1 for these events.

Another approach to study the influence of CYP2E1 on ALD involves the use of CYP2E1 inhibitors. Chlormethiazole is a specific CYP2E1 inhibitor [75] and has a pronounced inhibitory effect on ALD in the intra-gastric alcohol rat model [76]. Similar experiments were also conducted using diallyl sulfide and phenylethyl isothiocyanate as CYP2E1 inhibitors demonstrating a protective effect against hydroxyradical formation from ethanol and lipid peroxidation and inhibition of some pathological scores [77,78].

Together, these studies indicate a significant contribution of alcohol-dependent induction of CYP2E1 for the development of ALD [79]. The major cause is its ability to increase ROS formation after ethanol treatment, but other factors controlling the redox properties of the liver also contribute to the observed pathology.

### Intestinal CYP2E1 in ALD

In addition to causing gut dysbiosis, alcohol increases CYP2E1 levels and nitroxidative stress in the intestinal epithelium similarly as in the liver [80,81,82,83,84]. This causes intestinal leakiness followed by increased circulating endotoxin levels [82,85,86,87]. Endotoxin can initiate a hepatic necroinflammatory cascade starting from increased levels of NF-κB and release of inflammatory cytokines, such as TNF-α by Kupffer cells [85,88,89]. These results suggest a role of intestinal protein nitration in mediating alcohol-induced gut leakiness and subsequent hepatic injury in a CYP2E1-dependent manner. Even though fatty acids induce CYP2E1 in liver, as discussed later in this review, it is unclear whether this holds for intestinal CYP2E1 and increases gut leakiness playing a role in the development of NAFLD. NASH patients exhibit increased gut leakiness [90] and dysbiosis (reviewed in [91]) and gut inflammation and dysbiosis augments hepatic inflammation and fibrogenesis in mouse NASH models. However, the role of intestinal CYP2E1 and oxidative stress for gut leakiness was not tested [90,92,93]. It has been hypothesized that ethanol produced by microbiota fermenting dietary sugars could cause dysbiosis, increased CYP2E1 levels and nitroxidative stress in NAFLD patients (reviewed in [91]) and maybe endotoxin levels are thus lower in patients with alcoholic cirrhosis compared with non-alcoholic cirrhosis [87].

## 5. CYP2E1 in NAFLD

NAFLD has been known for over 40 years; despite this, the underlying mechanisms remain poorly understood [94]. NAFLD refers to a continuum of liver diseases beginning with non-alcoholic fatty liver (NAFL), an accumulation of hepatic lipids not explained by alcohol consumption. In some cases, this can progress towards non-alcoholic steatohepatitis (NASH), fibrosis and cirrhosis and eventually HCC. Most NAFLD patients are obese and exhibit mild systemic inflammation, which induces insulin resistance and plays a role in the mechanism of liver damage [95,96,97,98]. NASH encompasses varying degrees of liver injury and is now recognized as the hepatic component of metabolic syndrome [99,100,101,102].

Liver cirrhosis is part of alcoholic steatohepatitis (ASH) and NASH, for which the only curative solution is often liver transplantation, and can progress to HCC in 3–4% of cases. The major causes of liver fibrosis are: hepatitis C infection (33%), alcohol (30%) and NASH (23%) [103]. The individual risks for liver cirrhosis caused by NASH and alcohol are largely determined by genetic risk factors, with polymorphisms in *PNPLA3* and *TM6SF2* and also *MBOAT7, HSD17B13* and *PCKS7* being the major common genetic determinants [104].

The multiple parallel hit model aims to explain the initiation and progression of NAFLD and has, in recent years, superseded the more simplistic two-hit model [101,105]. It suggests that multiple concurrent environmental and genetic insults, such as insulin resistance, oxidative stress-induced mitochondrial dysfunction, ER stress, endotoxin-induced TLR4-dependent release of inflammatory cytokines, and free fatty acid (FFA) accumulation combine to produce cell death and damage [106]. Oxidative stress mediated by ROS likely plays a primary role as the initiator of hepatic and extrahepatic damage and can cause damage in myriad ways by peroxidation of cellular macromolecules [107]. Oxidative stress can lead to lipid accumulation both directly and indirectly, most simplistically, ROS can peroxidate cellular lipids. Presence of these peroxidated lipids increases post-translational degradation of ApoB, preventing hepatocellular lipid export and leading to lipid accumulation. Alternatively, ROS can directly peroxidate proteins such as ApoB, directly preventing their function and producing a similar effect [108].

**Figure 4:**
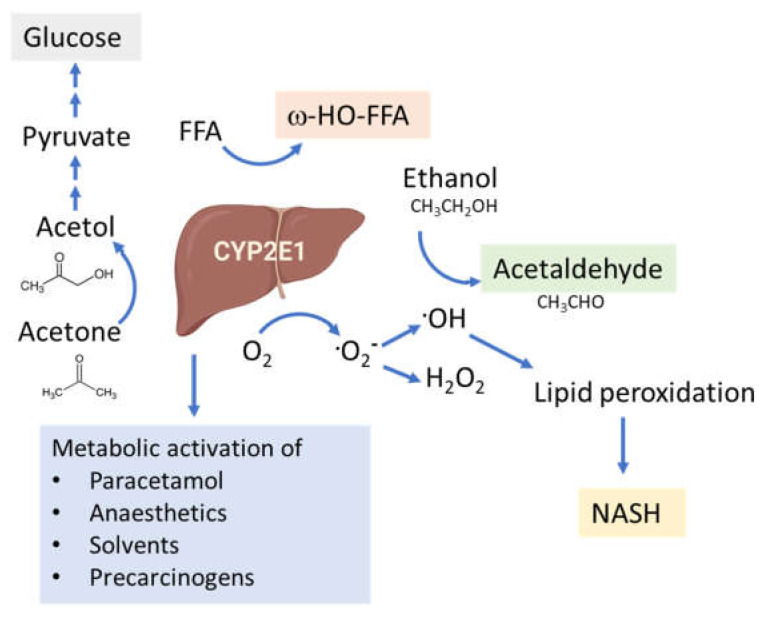
Functions of the CYP2E1 enzyme. CYP2E1 has a role in normal physiological homeostasis by metabolizing endogenous and exogenous compounds. Major endogenous functions are ω-hydroxylation of FFA and oxidation of acetone to precursors of gluconeogenesis. Increased amounts of CYP2E1 results in increased lipid peroxidation and ROS production and is associated with the progression of NAFLD to NASH.

The connection between CYP2E1 and NASH was first suggested by Weltman et al., in 1996, where elevated levels of CYP2E1 were observed in steatosis and NASH patients, particularly in the centrilobular region [109] corresponding to the site of maximal hepatocellular injury in NASH [41,110]. This connection is based on the high propensity of CYP2E1 to generate ROS, even in the absence of substrates (Figure 4). In addition, CYP2E1 levels are elevated in obesity, steatosis and NASH in both humans and rodents [111,112,113].

### 5.1. CYP2E1 Links Insulin Resistance and NAFLD

The increased circulating levels of ketone bodies and fatty acids observed in obesity, steatosis and NASH may induce CYP2E1 and insulin resistance [66,106,114,115]. In addition, *Cyp2e1* knockout mice are protected against high-fat diet-induced obesity and insulin resistance and the production of proinflammatory cytokines in adipose tissue was prevented [116]. CYP2E1 was recently shown to be linked to insulin resistance via the anti-apoptotic protein Bax inhibitor-1, which plays an important role in the regulation of CYP2E1 [117,118]. Furthermore, the repressive effects of insulin on CYP2E1 levels are lost in insulin resistance, commonly associated with NAFLD and NASH [106,115].

### 5.2. The Role of CYP2E1 in Hepatic Lipid Accumulation

Experiments preventing the degradation of CYP2E1 in mice by ablation of E3 ubiquitin ligase have demonstrated that the elevation of CYP2E1 alone in this model is insufficient to induce NASH. Concurrent elevation of CYP2E1 and induction of hepatic lipid accumulation from enhanced liver fat-production or ingestion of a high fat/high carbohydrate diet is required to induce NASH-like symptoms [32]. Conversely, CYP2E1 stabilization with CHIP-knockout in mice was associated with increased functional activity together with microvesicular fat accumulation and increased lipid peroxidation by activation of the hepatic JNK-cascade [119]. This occurred even though the mice were fed with a non-fat/carbohydrate-enriched diet, suggesting that overexpression of hepatic CYP2E1 and consequent oxidative stress was sufficient for NASH development [119]. This occurred even though the mice were fed with a non-fat/carbohydrate-enriched diet, suggesting that overexpression of hepatic CYP2E1 and consequent oxidative stress was sufficient for NASH development. Further research is required to elucidate the complexities of the role of CYP2E1 in hepatic lipid accumulation.

### 5.3. Complementary Roles of CYP4A and CYP2E1 in Lipid Oxidation through PPARα

Animal studies have suggested complementary metabolic roles of CYP2E1 and CYP4A in lipid oxidation and the production of oxidative stress [66,120]. Besides CYP2E1, only CYP4A11 is responsible for the ω-hydroxylation of medium-length chain carboxylic acids in humans [121,122]. Silencing of *CYP2E1* in mice fed a methionine-choline deficient diet increased CYP4A levels and the animals displayed elevated lipid peroxidation and NASH-like symptoms [120]. In addition, antibodies against CYP2E1 inhibited lipid peroxidation in microsomes from wild-type mice, but antibodies against CYP4A had little effect [120]. The inverse was found with microsomes from *Cyp2e1* knockout mice where antibodies against CYP4A blocked lipid peroxidation, whilst antibodies against CYP2E1 had no effect. Thus, it could be concluded that CYP4 serves as an initiator or catalyst of oxidative stress as a complimentary pathway to CYP2E1.

In humans, CYP4A11 is elevated in NAFLD patients [123]. For this reason, it is more difficult to study the role of CYP2E1 alone in oxidative stress. Structure-activity relationship studies on CYP4A11 and its orthologs and CYP2E1 have confirmed similarity in their potential to be inhibited [122]. This was further tested using a strong inhibitor of CYP4A as a new class of drug candidate in targeting CYP2E1. These drugs, 12-imidazolyl-1-dodecanol and 1-imidazolyldodecane, are competitive inhibitors of the active site of CYP2E1 and restored intracellular redox balance via reduction of ROS and lipid peroxidation in vitro as well as in rats fed with high-fat diet or alcohol. Such ω-imidazolyl-alkyl derivatives inhibiting CYP2E1 may serve as a possible new therapeutic approach to NAFLD and especially to NASH [124,125]. However, it is unclear whether dual inhibition of CYP4A and CYP2E1 or only inhibition of CYP2E1 is required for these effects.

De novo lipogenesis and fatty acid oxidation are implicated in NAFLD pathogenesis, although lipid uptake, storage and export also play a role [126,127]. Fatty acid oxidation is a cyclic process in which fatty acids are shortened, releasing acetyl-CoA after each cycle [128,129]. One of the main regulators of this process is the peroxisome proliferator-activated receptor-α (PPARα) [130], However, other factors are also involved in fine-tuning the process. Both CYP4A and CYP2E1 enzymes appear to interact with the PPARα pathway. Thus, CYP4A genes are partly controlled by PPARα; Kroetz et al., showed that the induction of CYP4A in diabetes and under starvation conditions in rats was dependent on PPARα expression [131]. Centrilobular fat accumulation and upregulation of PPARα and PPARα-mediated pathways in *Cyp2e1*-null mice fed with ethanol indicates an interplay between CYP2E1 and PPARα-mediated fatty acid homeostasis [132]. Increased mitochondrial fat oxidation was speculated to be due to the PPARα mediated induction of carnitine palmitoyl transferase I. These data suggest that CYP2E1 and ethanol can regulate PPARα-mediated fatty acid homeostasis, but PPARα only becomes important when the CYP2E1 level is low in high-fat conditions. The physiological relevance of this scenario is dubious since high-fat conditions induce CYP2E1 expression.

Abdelmegeed et al., showed a significant fat-induced increase in mitochondrial CYP2E1 in both wild type and PPARα-null mice [133]. PPARα-deficient mice showed greater mitochondrial dysfunction, regardless of diet, evidenced by reduced expression of mitochondrial 3-ketoacyl-CoA thiolase. However, the resultant increase in oxidative stress was more prominent in *PPARα*-null mice, which exhibited higher levels of CYP2E1 than the corresponding wild-type mice. These data suggest that mitochondrial CYP2E1 might play a role, at least partially, in mediating high-fat induced NASH development in *PPARα*-null mice. Taken together, even though these studies show interplay between CYP2E1, CYP4A and PPARα, all of which play a role in NASH development and progression, the clinical relevance has not yet been demonstrated.

### 5.4. Possible Role of Mitochondrial CYP2E1 in NAFLD

Different CYP2E1 isoforms exist in several cell compartments including the ER and mitochondria as previously mentioned [82,134]. In recent years, the role of non-ER-based CYP2E1 in the development and progression of NAFLD has gathered interest. Currently, the differences, if any, between isoforms in terms of induction or substrates is not well understood. Mitochondrial CYP2E1 is expressed at approximately 30% of the level of the microsomal CYP2E1 under basal conditions in rats and is associated with elevated mitochondrial oxidative stress [135]. It is present in two forms, one highly phosphorylated form mediated via cAMP-dependent protein kinase A, and one shortened amino terminal-truncated form [19,20,135]. The regulation of these modifications is unclear, however, they are hypothesized to cause conformational changes and altered interactions with molecular chaperones and signal recognition particles, directing the CYP2E1 to the mitochondria.

Differences in mitochondrial CYP2E1 from its microsomal counterpart in both alcoholic fatty liver disease (AFLD) and NAFLD have been reported. A few studies have suggested that mitochondrial CYP2E1 is a major source of alcohol and drug-induced oxidative stress [112,136,137]. Mitochondrial CYP2E1 may be more responsible for the damage to mitochondrial function and membrane and may contribute to the biochemical and toxicological effects which were previously ascribed to CYP2E1 in the ER. Whether CYP2E1 in both ER and mitochondria work simultaneously or sequentially, and whether mitochondrial CYP2E1 exerts more pronounced effects on mitochondrial dysfunction in AFLD and NAFLD, is unclear due to lack of specific inhibitors [82]. Mitochondrial CYP2E1 may have a longer half-life than CYP2E1 in the ER, possibly due to unavailable ER degradation by the ubiquitin-proteasome pathway [138].

### 5.5. Kupffer Cells and CYP2E1 in NAFLD

The progression of NAFLD to NASH is characterized by increased inflammation and fibrosis. Therefore, liver-resident immune cells, such as Kupffer cells, are implicated. Their activation is driven by several factors, including cytokines and cell death [139]. Macrophages may be activated when hepatocytes die as a result of ROS and lipid-induced stress, their contents are released and detected by macrophages as damage-associated molecular patterns such as HMGB1 and caspase-cleaved keratin 18 [140]. CYP2E1 is also inducible in Kupffer cells [16,17,18] and the lipid peroxidation product 4-hydroxynonenal upregulates transforming growth factor-β expression in macrophages, causing further inflammation [141]. Macrophages with stably increased CYP2E1 expression (murine RAW 264.7 macrophages transfected with *CYP2E1*) displayed increased levels of CD14/Toll-like receptor 4, NADPH oxidase and H_2_O_2_, accompanied by activation of ERK1/2, p38, and NF-κB in [142]. Apart from mitochondria-derived ROS, NADPH oxidase 2 (NOX2) activation in liver-infiltrating macrophages has also been reported to contribute to oxidative stress-induced liver damage in NAFLD [143]. On the other hand, the amount of CYP2E1 detected in rat Kupffer cells was 10-fold lower than in hepatocytes [142] and, taking into consideration the comparative number of different liver cell types, the hepatocyte localization of CYP2E1 will be of utmost importance for causing oxidative stress.

### 5.6. CYP2E1-Mediated ROS Production and Lipid Peroxidation

As previously mentioned, ROS can be produced from many cell systems including the mitochondrial respiratory chain [144], the cytochromes P450 [145], and oxidative enzymes [146]. When compared with other cytochromes P450, CYP2E1 possesses a remarkably high NADPH oxidase activity, resulting in significant production of ROS, such as hydrogen peroxide, superoxide anion radicals and hydroxyl radicals in the presence of iron catalysts [49,65,67,82]. ROS production from CYP2E1 causes a free radical chain reaction with unsaturated fatty acids generating toxic lipid intermediates, a reaction magnified by the presence of free iron. Such lipid peroxidation products, e.g., the biologically active aldehydes, hydroxynonenal, 4-hydoxyhydroperoxy-2-nonenal and malondialdehyde, can also modify the integrity of cellular membranes and damage proteins and DNA [141,147].

ROS-mediated activation of the JNK pathway can interfere with insulin sensitivity through phosphorylation of IRS-1 and IRS-2 and impair glycogenesis via action on GSK3, leading to increased gluconeogenesis [32,148]. In addition, ROS increase the expression of several cytokines, including transforming growth factor-β, interleukin 8, tumor necrosis factor-α and Fas ligand [149,150,151,152]. Both cytokines and lipid peroxidation products may act together to trigger the diverse lesions of NASH [153]. By-products of ROS-induced damage, such as 4-hydroxynonenal and 3-nitrotyrosine, are significantly increased in the plasma and liver, respectively in NAFLD and NASH patients [134,154]. CYP2E1 is crucial in this regard as the production of ROS when it is induced, e.g., in response to FFAs, underlies much of this process.

In vivo data on the role of CYP2E1 in lipid peroxidation comes from rodent studies [74,78] and from the observation of CYP2E1 induction in human liver in NASH [109]. Using CYP2E1 inhibitors, Morimoto et al., found a relationship between CYP2E1 and lipid peroxidation in rats with ALD [78]. Besides increased lipid peroxidation, CYP2E1 was found to increase hepatic nitroxidative stress [77]. Abdelmegeed et al., used young and aged female wild-type and *Cyp2e1*-null mice to show that aged wild type mice had increased hepatic fibrosis, levels of hepatic hydrogen peroxide, lipid peroxidation compared with *Cyp2e1*-null mice [74]. These data are consistent with a role of CYP2E1 in development of liver fibrosis. There may also be a relationship between CYP2E1 and mitochondrial dysfunction due to mitochondrial CYP2E1-mediated ROS production [137]. In addition, lower ROS detoxification and lipid peroxidation-derived reactive aldehydes play a role in mitochondrial stress associated with NAFLD development and progression [155,156]. This can trigger a vicious cycle, further increasing ROS generation through abnormal electron leakage.

Together, these studies largely agree that CYP2E1-mediated increased nitroxidative radicals, lipid peroxidation, and post-translational protein modifications are the main mechanisms by which CYP2E1 likely plays a prominent role in NAFLD development and progression [82]. Expressed simply, CYP2E1 is a major source of hepatic ROS. The action of ROS from CYP2E1 and other sources can induce lipid peroxidation and cause other non-specific damage to cellular macromolecules. Therefore, conditions that induce the expression of CYP2E1 will also increase the production of ROS and ROS-related damage.

The link between oxidative stress and the development and progression of ALD and NAFLD has led to the study of the potential of antioxidants for prevention or treatment for these diseases using either Mediterranean diet or drugs. Clinical trials have shown that vitamin E can ameliorate NAFLD by attenuating oxidative stress and inflammation (reviewed in [157,158]); however, the safety of prolonged vitamin E use is uncertain [159,160,161]. In addition to vitamin E, flavonoids have anti-inflammatory and antioxidant properties and reduce the expression of CYP2E1 (reviewed in [162]). For instance, quercetin and several saponins, alkaloids, terpenoids and polyphenols have been tested in vivo and in vitro and many have shown promise for NASH treatment (reviewed in [163]). Unfortunately, these effects have not persisted under clinical trial conditions thus far.

## 6. In Vitro Models for Examining the Role of CYP2E1 and FFA on Liver Fibrosis and Damage

The liver is composed of hepatocytes (~60%) and non-parenchymal cells (NPCs) (~40%). The main types of NPCs are stellate cells, Kupffer cells and sinusoidal endothelial cells. NAFLD and NASH are not formed solely by the action of hepatocytes but are rather the results of complex interactions between the NPCs, hepatocytes and external factors. For this reason, models with only hepatocytes or hepatocyte-like cells cannot accurately recapitulate NAFLD development and progression compared with heterocellular models. However, by comparing the monocellular and heterocellular NASH models the role of NPCs in the development and progression of NAFLD can be studied [164].

In vitro models can be divided into groups based on the cell types and culture methods employed. Many recent advances in in vitro models for NAFLD and liver toxicity in general use 3D cultures, often due to the increased physiological relevance these models can offer. There are multiple 3D culture techniques ranging from matrix-based systems to suspension cultures, both with and without liquid flow. In addition, tissue-on-a-chip models have been developed and show great promise. Some advances in modelling NAFLD were recently reviewed by Soret et al. [165].

**Figure 5:**
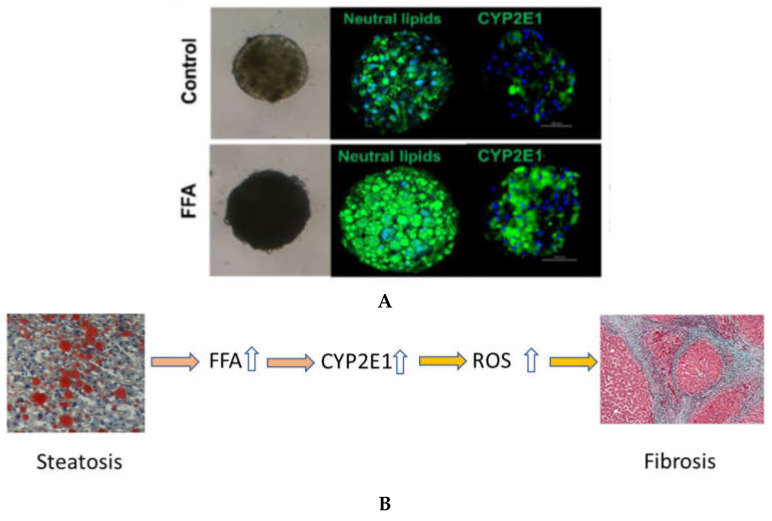
Suggested link between steatosis and liver fibrosis mediated by elevated levels of CYP2E1 caused by increase production of fat. (**A**) Exposure to FFA in human 3D liver spheroids causes elevated levels of lipids and increase expression of CYP2E1 (From [164]). (**B**) Proposed involvement of CYP2E1 in mediating increased liver fibrosis in response to elevated levels of hepatic lipids in steatosis. Upwards arrows represent an increased number of molecules or greater expression. Figure modified from [163].

Human primary hepatocytes (PHH) dedifferentiate in two-dimensional culture systems precluding their useful application to long-term experiments, such as the development of NAFLD. Compared with traditional monolayer cell culture models, 3D Spheroid models have been shown to more accurately mimic the in vivo environment [52]. PHH 3D spheroids maintain tissue-like architecture, cell–cell interactions and hepatic phenotype and can be used to model the onset of NAFLD (Figure 5) [164,166,167,168]. Using such a 3D PHH spheroid model with NPCs, we found that NPCs and FFA induce CYP2E1 on the mRNA and protein level [164]. In some donors, heterocellular spheroids showed elevated CYP2E1 protein expression in the presence of NPCs only, without added FFA. This might be indicative of the fact that increased lipid levels during steatosis might stimulate induction of liver fibrosis by the subsequent induction of CYP2E1 causing an increase ROS. The contribution of CYP2E1 in NASH may indeed be of importance, but more studies are needed in this topic.

## 7. Conclusions

It is evident that CYP2E1 has important functions for both lipid and glucose homeostasis as well as being an important enzyme in toxicology. The enzyme is highly conserved with essentially no functionally different genetic variants, emphasizing its important endogenous functions, many of which may still be unknown. The toxicologically relevant functions are to a great extent related to the high-spin nature of the iron in the enzyme, allowing effective reduction of dioxygen and other compounds in the absence of bound substrate, as well as both reductive and oxidative radical formation by the enzyme. The enzyme action is important for generation of ALD and NASH and it is likely that the link between steatosis and NASH could to some extent be explained by the fact that excess lipids highly induce the hepatic levels of CYP2E1, thus resulting in ROS stress and increased lipid peroxidation, key events for development of NASH.

Although much has been learned, there are still many factors to consider for the future. CYP2E1 resides mainly in the ER of hepatocytes where it takes part in the metabolism of fatty acids, acetone and other endogenous compounds. The function of mitochondrial CYP2E1, if any, is unknown.

The contribution of CYP2E1 to elimination of ethanol is still controversial and conclusions differ greatly between authors. In addition, the relationship between the specific and nonspecific CYP2E1-mediated oxyradical-mediated oxidation of ethanol is unclear. CYP2E1 expression is elevated following ethanol treatment, but the rate of ethanol oxidation is very low, as compared with ADH. Furthermore, ADH is strongly induced by high ethanol levels and might represent the major component for adaptive ethanol oxidation.

The role of CYP2E1 for development of NASH is still unclear. Further experimentation is necessary to directly show the role of CYP2E1 in enhanced production of mediators activating stellate cells to produce profibrotic cytokines and, indeed, the effect of CYP2E1 on activation of liver endothelial cells has not been described. The elevation of CYP2E1 following lipid treatment may have anti-steatotic effects because of higher rates of lipid degradation. Inhibitors of CYP2E1 have been shown to be effective for the development of ALD, but must also be examined for NASH production.

## Abbreviations

3D	three dimensional
ADH	alcohol dehydrogenase
AFLD	alcoholic fatty liver disease
ALD	alcoholic liver disease
ASH	alcoholic steatohepatitis
CDE	chlorodifluoroethylene
CTE	chlorotrifluoroethane
CYP2E1	cytochrome P450 2E1
ER	endoplasmic reticulum
FFA	free fatty
HCC	hepatocellular carcinoma
hPSC	human pluripotent stem cell
NADPH	nicotinamide adenine dinucleotide phosphate acid
NAFL	nonalcoholic fatty liver
NAFLD	nonalcoholic fatty liver disease
NASH	nonalcoholic steatohepatitis
NPC	non-parenchymal cell
PHH	human primary hepatocyte
POR	P450 reductase
ROS	reactive oxygen species
TFA	trifluoroacetic acid

## References

[1] Shigeta Y., Nomura F., Iida S., Leo M.A., Felder M.R., Lieber C.S. (1984). Ethanol metabolism in vivo by the microsomal ethanol-oxidizing system in deermice lacking alcohol dehydrogenase (ADH). Biochem. Pharmacol..

[2] Teschke R., Hasumura Y., Lieber C.S. (1975). Hepatic microsomal alcohol-oxidizing system. Affinity for methanol, ethanol, propanol, and butanol. J. Biol. Chem..

[3] Morgan E.T., Devine M., Skett P. (1981). Changes in the rat hepatic mixed function oxidase system associated with chronic ethanol vapor inhalation. Biochem. Pharmacol..

[4] Ingelman-Sundberg M., Johansson I. (1981). The mechanism of cytochrome P-450-dependent oxidation of ethanol in reconstituted membrane vesicles. J. Biol. Chem..

[5] I Cederbaum A., Dicker E., Gutteridge J.M., Smith A., A Clejan L., Groebler L.K., Liu J., Shanu A., Codd R., Witting P.K. (1983). Inhibition of microsomal oxidation of alcohols and of hydroxyl-radical-scavenging agents by the iron-chelating agent desferrioxamine. Biochem. J..

[6] Teschke R., Moreno F., Petrides A.S. (1981). Hepatic microsomal ethanol oxidizing system (MEOS): Respective roles of ethanol and carbohydrates for the enhanced activity after chronic alcohol consumption. Biochem. Pharmacol..

[7] Koop D.R., Morgan E., E Tarr G., Coon M.J. (1982). Purification and characterization of a unique isozyme of cytochrome P-450 from liver microsomes of ethanol-treated rabbits. J. Biol. Chem..

[8] Ingelman-Sundberg M., Hagbjork A.L. (1982). On the significance of the cytochrome P-450-dependent hydroxyl radical-mediated oxygenation mechanism. Xenobiotica.

[9] Khani S.C., Zaphiropoulos P., Fujita V.S., Porter T.D., Koop D.R., Coon M.J. (1987). cDNA and derived amino acid sequence of ethanol-inducible rabbit liver cytochrome P-450 isozyme 3a (P-450ALC). Proc. Natl. Acad. Sci. USA.

[10] Nebert D.W., Nelson D.R., Coon M.J., Estabrook R.W., Feyereisen R., Fujii-Kuriyama Y., Gonzalez F.J., Guengerich F.P., Gunsalus I.C., Johnson E.F. (1991). The P450 Superfamily: Update on New Sequences, Gene Mapping, and Recommended Nomenclature. DNA Cell Biol..

[11] Umeno M., McBride O.W., Yang C.S., Gelboin H.V., Gonzalez F.J. (1988). Human ethanol-inducible P450IIE1: Complete gene sequence, promoter characterization, chromosome mapping, and cDNA-directed expression. Biochemistry.

[12] Gaedigk A., Sangkuhl K., Whirl-Carrillo M., Twist G.P., Klein T.E., Miller N.A. (2019). The PharmVar Steering Committee The Evolution of PharmVar. Clin. Pharmacol. Ther..

[13] Tanaka E., Terada M., Misawa S. (2000). Cytochrome P450 2E1: Its clinical and toxicological role. J. Clin. Pharm. Ther..

[14] Ronis M.J.J., Lindros K.O., Ingelman-Sundberg M. (1996). The CYP2E subfamily. Cytochromes P.

[15] Ma H., Chen S., I Zheng K., Yu Y., Wang X., Tang L., Li G., Rios R.S., Huang O., Zheng X. (2021). TA allele of rs2070673 in the CYP2E1 gene is associated with lobular inflammation and nonalcoholic steatohepatitis in patients with biopsy-proven nonalcoholic fatty liver disease. J. Gastroenterol. Hepatol..

[16] Koop D.R., Chernosky A., Brass E.P. (1991). Identification and induction of cytochrome P450 2E1 in rat Kupffer cells. J. Pharmacol. Exp. Ther..

[17] Koivisto T., Mishin V.M., Mak K.M., Cohen P.A., Lieber C.S. (1996). Induction of Cytochrome P-4502E1 by Ethanol in Rat Kupffer Cells. Alcohol. Clin. Exp. Res..

[18] Ye Q., Wang X., Wang Q., Xia M., Zhu Y., Lian F., Ling W. (2013). Cytochrome P4502E1 inhibitor, chlormethiazole, decreases lipopolysaccharide-induced inflammation in rat Kupffer cells with ethanol treatment. Hepatol. Res..

[19] Neve E.P.A., Ingelman-Sundberg M. (2000). Molecular Basis for the Transport of Cytochrome P450 2E1 to the Plasma Membrane. J. Biol. Chem..

[20] Neve E.P., Ingelman-Sundberg M. (1999). A soluble NH2-terminally truncated catalytically active form of rat cytochrome P450 2E1 targeted to liver mitochondria. FEBS Lett..

[21] Lieber C.S. (1997). Cytochrome P-4502E1: Its physiological and pathological role. Physiol. Rev..

[22] Loeper J., Descatoire V., Maurice M., Beaune P., Feldmann G., Larrey D., Pessayre D. (1990). Presence of functional cytochrome P-450 on isolated rat hepatocyte plasma membrane. Hepatology.

[23] Loeper J., Descatoire V., Maurice M., Beaune P., Belghiti J., Houssin D., Ballet F., Feldmann G., Guengerich F., Pessayre D. (1993). Cytochromes P-450 in human hepatocyte plasma membrane: Recognition by several autoantibodies. Gastroenterology.

[24] Neve E.P., Eliasson E., Pronzato M.A., Albano E., Marinari U., Ingelman-Sundberg M. (1996). Enzyme-Specific Transport of Rat Liver Cytochrome P450 to the Golgi Apparatus. Arch. Biochem. Biophys..

[25] Koop D.R. (1992). Oxidative and reductive metabolism by cytochrome P450 2E1. FASEB J..

[26] Massart J., Begriche K., Fromenty B. (2021). Cytochrome P450 2E1 should not be neglected for acetaminophen-induced liver injury in metabolic diseases with altered insulin levels or glucose homeostasis. Clin. Res. Hepatol. Gastroenterol..

[27] Seitz H.K. (2020). The role of cytochrome P4502E1 in the pathogenesis of alcoholic liver disease and carcinogenesis. Chem. Interact..

[28] Seitz H.K., Mueller S. (2014). Alcohol and Cancer: An Overview with Special Emphasis on the Role of Acetaldehyde and Cytochrome P450 2E1. Adv. Exp. Med. Biol..

[29] Johansson I., Eliasson E., Norsten C., Ingelman-Sundberg M. (1986). Hydroxylation of acetone by ethanol- and acetone-inducible cytochrome P-450 in liver microsomes and reconstituted membranes. FEBS Lett..

[30] Song B., Cederbaum A.I. (1996). Ethanol-Inducible Cytochrome P450 (CYP2E1): Biochemistry, Molecular Biology and Clinical Relevance: 1996 Update. Alcohol. Clin. Exp. Res..

[31] Gonzalez F.J. (2005). Role of cytochromes P450 in chemical toxicity and oxidative stress: Studies with CYP2E1. Mutat. Res. Mol. Mech. Mutagen..

[32] Correia M.A., Kwon D. (2020). Why Hepatic CYP2E1-Elevation by Itself Is Insufficient for Inciting NAFLD/NASH: Inferences from Two Genetic Knockout Mouse Models. Biology.

[33] Badger T.M., Ronis M.J.J., Ingelman-Sundberg M., Hakkak R. (1995). Inhibition of CYP2E1 Activity does not Abolish Pulsatile Urine Alcohol Concentrations During Chronic Alcohol Infusions. JBIC J. Biol. Inorg. Chem..

[34] Porubsky P.R., Meneely K.M., Scott E.E. (2008). Structures of Human Cytochrome P-450 2E1. J. Biol. Chem..

[35] Porubsky P.R., Battaile K., Scott E.E. (2010). Human Cytochrome P450 2E1 Structures with Fatty Acid Analogs Reveal a Previously Unobserved Binding Mode. J. Biol. Chem..

[36] Koop D.R., Crump B.L., Nordblom G.D., Coon M.J. (1985). Immunochemical evidence for induction of the alcohol-oxidizing cytochrome P-450 of rabbit liver microsomes by diverse agents: Ethanol, imidazole, trichloroethylene, acetone, pyrazole, and isoniazid. Proc. Natl. Acad. Sci. USA.

[37] Koop D.R., Tierney D.J. (1990). Multiple mechanisms in the regulation of ethanol-inducible cytochrome P450IIE1. BioEssays.

[38] Song B.J., Matsunaga T., Hardwick J.P., Park S.S., Veech R.L., Yang C.S., Gelboin H.V., Gonzalez F.J. (1987). Stabilization of Cytochrome P450j Messenger Ribonucleic Acid in the Diabetic Rat. Mol. Endocrinol..

[39] Yun Y.P., Casazza J.P., Sohn D.H., Veech R.L., Song B.J. (1992). Pretranslational activation of cytochrome P450IIE during ketosis induced by a high fat diet. Mol. Pharmacol..

[40] Novak R.F., Woodcroft K.J. (2000). The alcohol-inducible form of cytochrome P450 (CYP2E1): Role in toxicology and regulation of expression. Arch. Pharml. Res..

[41] Johansson I., Lindros K.O., Eriksson H., Ingelman-Sundberg M. (1990). Transcriptional control of CYP2E1 in the perivenous liver region and during starvation. Biochem. Biophys. Res. Commun..

[42] Yap C.G., Zaini A., Othman I. (2016). Targeted CYP2E1 quantification and its correlation to currently acceptable clinical biochemical indices. J. Biol. Res..

[43] Lee S.S., Buters J., Pineau T., Fernandez-Salguero P.M., Gonzalez F.J. (1996). Role of CYP2E1 in the Hepatotoxicity of Acetaminophen. J. Biol. Chem..

[44] Johansson I., Ekstroem G., Scholte B., Puzycki D., Joernvall H., Ingelman-Sundberg M. (1988). Ethanol-, fasting-, and aceton-inducible cytochromes P-450 in rat liver: Regulation and characteristics of enzymes belonging to the IIB and IIE gene subfamilies. Biochemistry.

[45] Koop D.R., Coon M.J. (1984). Purification of liver microsomal cytochrome P-450 isozymes 3a and 6 from imidazole-treated rabbits. Evidence for the identity of isozyme 3a with the form obtained by ethanol treatment. Mol. Pharmacol..

[46] Song B.J., Gelboin H.V., Park S.S., Yang C.S., Gonzalez F.J. (1986). Complementary DNA and protein sequences of ethanol-inducible rat and human cytochrome P-450s. Transcriptional and post-transcriptional regulation of the rat enzyme. J. Biol. Chem..

[47] Eliasson E., Johansson I., Ingelman-Sundberg M. (1990). Substrate-, hormone-, and cAMP-regulated cytochrome P450 degradation. Proc. Natl. Acad. Sci. USA.

[48] Eliasson E., Mkrtchian S., Ingelman-Sundberg M. (1992). Hormone- and substrate-regulated intracellular degradation of cytochrome P450 (2E1) involving MgATP-activated rapid proteolysis in the endoplasmic reticulum membranes. J. Biol. Chem..

[49] Lu Y., Cederbaum A.I. (2008). CYP2E1 and oxidative liver injury by alcohol. Free Radic. Biol. Med..

[50] Wang Y., Guan S., Acharya P., Koop D.R., Liu Y., Liao M., Burlingame A.L., Correia M.A. (2011). Ubiquitin-dependent Proteasomal Degradation of Human Liver Cytochrome P450 2E1. J. Biol. Chem..

[51] Ronis M.J.J., Johansson I., Hultenby K., Lagercrantz J., Glaumann H., Ingelman-Sundberg M. (1991). Acetone-regulated synthesis and degradation of cytochrome P4502E2 and cytochrome P4502B1 in rat liver. JBIC J. Biol. Inorg. Chem..

[52] Bell C.C., Hendriks D., Moro S.M.L., Ellis E., Walsh J., Renblom A., Puigvert L.F., Dankers A.C.A., Jacobs F., Snoeys J. (2016). Characterization of primary human hepatocyte spheroids as a model system for drug-induced liver injury, liver function and disease. Sci. Rep..

[53] Owen O.E., Patel M.S., Block B.S.B., Kreulen T.H., Reichle F.A., Mozzoli M.A. (1976). Gluconeogenesis in normal, cirrhotic, and diabetic humans. Gluconeogenesis: Its Regulation in Mammalian Species.

[54] Ingelman-Sundberg M., Johansson I. (1984). Mechanisms of hydroxyl radical formation and ethanol oxidation by ethanol-inducible and other forms of rabbit liver microsomal cytochromes P-450. J. Biol. Chem..

[55] Cederbaum A.I., Dicker E., Cohen G. (1980). Role of hydroxyl radicals in the iron-ethylenediaminetetraacetic acid mediated stimulation of microsomal oxidation of ethanol. Biochemistry.

[56] Johansson I., Ingelman-Sundberg M. (1985). Carbon tetrachloride-induced lipid peroxidation dependent on an ethanol-inducible form of rabbit liver microsomal cytochrome P-450. FEBS Lett..

[57] Safari S., Motavaf M., Siamdoust S.A.S., Alavian S.M. (2014). Hepatotoxicity of Halogenated Inhalational Anesthetics. Iran. Red Crescent Med. J..

[58] Spracklin D.K., Emery M.E., Thummel K.E., Kharasch E.D. (2003). Concordance between trifluoroacetic acid and hepatic protein trifluoroacetylation after disulfiram inhibition of halothane metabolism in rats. Acta Anaesthesiol. Scand..

[59] Clot P., Albano E., Eliasson E., Tabone M., Aricò S., Israel Y., Moncada C., Sundberg M.I.- (1996). Cytochrome P4502E1 hydroxyethyl radical adducts as the major antigen in autoantibody formation among alcoholics. Gastroenterology.

[60] Spracklin D.K., Hankins D.C., Fisher J.M., E Thummel K., Kharasch E.D. (1997). Cytochrome P450 2E1 is the principal catalyst of human oxidative halothane metabolism in vitro. J. Pharmacol. Exp. Ther..

[61] Kharasch E., Hankins D., Mautz D., Thummel K. (1996). Identification of the enzyme responsible for oxidative halothane metabolism: Implications for prevention of halothane hepatitis. Lancet.

[62] Knockaert L., Berson A., Ribault C., Prost P.-E., Fautrel A., Pajaud J., Lepage S., Lucas-Clerc C., Bégué J.-M., Fromenty B. (2011). Carbon tetrachloride-mediated lipid peroxidation induces early mitochondrial alterations in mouse liver. Lab. Investig..

[63] Mann R.E., Smart R.G., Govoni R. (2003). The epidemiology of alcoholic liver disease. Alcohol Res. Health.

[64] Sakaguchi S., Takahashi S., Sasaki T., Kumagai T., Nagata K. (2011). Progression of Alcoholic and Non-alcoholic Steatohepatitis: Common Metabolic Aspects of Innate Immune System and Oxidative Stress. Drug Metab. Pharmacokinet..

[65] Leung T.-M., Nieto N. (2013). CYP2E1 and oxidant stress in alcoholic and non-alcoholic fatty liver disease. J. Hepatol..

[66] Robertson G., Leclercq I., Farrell G.C. (2001). Cytochrome P-450 enzymes and oxidative stress. Am. J. Physiol. Liver Physiol..

[67] Ekström G., Ingelman-Sundberg M. (1989). Rat liver microsomal NADPH-supported oxidase activity and lipid peroxidation dependent on ethanol-inducible cytochrome P-450 (P-450IIE1). Biochem. Pharmacol..

[68] Ingelman-Sundberg M., Johansson I., Yin H., Terelius Y., Eliasson E., Clot P., Albano E. (1993). Ethanol-inducible cytochrome P4502E1: Genetic polymorphism, regulation, and possible role in the etiology of alcohol-induced liver disease. Alcohol.

[69] Zhukov A., Ingelman-Sundberg M. (1999). Relationship between cytochrome P450 catalytic cycling and stability: Fast degradation of ethanol-inducible cytochrome P450 2E1 (CYP2E1) in hepatoma cells is abolished by inactivation of its electron donor NADPH–cytochrome P450 reductase. Biochem. J..

[70] Bell L.C., Guengerich F.P. (1997). Oxidation Kinetics of Ethanol by Human Cytochrome P450 2E1. J. Biol. Chem..

[71] Gorsky L.D., Koop D.R., Coon M.J. (1984). On the stoichiometry of the oxidase and monooxygenase reactions catalyzed by liver microsomal cytochrome P-450. Products of oxygen reduction. J. Biol. Chem..

[72] Morgan K., French S.W., Morgan T.R. (2002). Production of a cytochrome P450 2E1 transgenic mouse and initial evaluation of alcoholic liver damage. Hepatology.

[73] Butura A., Nilsson K., Morgan K., Morgan T.R., French S.W., Johansson I., Schuppe-Koistinen I., Ingelman-Sundberg M. (2009). The impact of CYP2E1 on the development of alcoholic liver disease as studied in a transgenic mouse model. J. Hepatol..

[74] Abdelmegeed M.A., Choi Y., Ha S.-K., Song B.-J. (2016). Cytochrome P450-2E1 promotes aging-related hepatic steatosis, apoptosis and fibrosis through increased nitroxidative stress. Free Radic. Biol. Med..

[75] Hu Y., Mishin V., Johansson I., Von Bahr C., Cross A., Ronis M.J., Badger T.M., Ingelman-Sundberg M. (1994). Chlormethiazole as an efficient inhibitor of cytochrome P450 2E1 expression in rat liver. J. Pharmacol. Exp. Ther..

[76] Gouillon Z.-Q., Lucas D., Li J., Hagbjork A.L., French B.A., Fu P., Fang C., Ingelman-Sundberg M., Donohue T.M., French S.W. (2000). Inhibition of ethanol-induced liver disease in the intragastric feeding rat model by chlormethiazole. Proc. Soc. Exp. Boil. Med..

[77] Albano E., Clot P., Morimoto M., Tomasi A., Ingelman-Sundberg M., French S.W. (1996). Role of cytochrome P4502E1-dependent formation of hydroxyethyl free radical in the development of liver damage in rats intragastrically fed with ethanol. Hepatology.

[78] Morimoto M., Hagbjork A.-L., Wan Y.-J.Y., Fu P.C., Clot P., Albano E., Ingelman-Sundberg M., French S.W. (1995). Modulation of experimental alcohol-induced liver disease by cytochrome P450 2E1 inhibitors. Hepatology.

[79] Seitz H., Neuman M. (2021). The History of Alcoholic Liver Disease: From an Unrecognized Disease to One of the Most Frequent Diseases in Hepatology. J. Clin. Med..

[80] Ballway J., Song B.-J. (2021). Translational Approaches with Antioxidant Phytochemicals against Alcohol-Mediated Oxidative Stress, Gut Dysbiosis, Intestinal Barrier Dysfunction, and Fatty Liver Disease. Antioxidants.

[81] Abdelmegeed M.A., Banerjee A., Jang S., Yoo S.-H., Yun J.-W., Gonzalez F.J., Keshavarzian A., Song B.-J. (2013). CYP2E1 potentiates binge alcohol-induced gut leakiness, steatohepatitis, and apoptosis. Free Radic. Biol. Med..

[82] Abdelmegeed M.A., Ha S.-K., Choi Y., Akbar M., Song B.-J. (2017). Role of CYP2E1 in Mitochondrial Dysfunction and Hepatic Injury by Alcohol and Non-Alcoholic Substances. Curr. Mol. Pharmacol..

[83] Tang Y., Forsyth C.B., Farhadi A., Rangan J., Jakate S., Shaikh M., Banan A., Fields J.Z., Keshavarzian A. (2009). Nitric Oxide-Mediated Intestinal Injury Is Required for Alcohol-Induced Gut Leakiness and Liver Damage. Alcohol. Clin. Exp. Res..

[84] Forsyth C.B., Voigt R.M., Shaikh M., Tang Y., Cederbaum A.I., Turek F.W., Keshavarzian A. (2013). Role for intestinal CYP2E1 in alcohol-induced circadian gene-mediated intestinal hyperpermeability. Am. J. Physiol. Liver Physiol..

[85] Keshavarzian A., Fields J. (2003). Alcoholic liver disease: Is it an “extraintestinal” complication of alcohol-induced intestinal injury?. J. Lab. Clin. Med..

[86] Tamai H., Kato S., Horie Y., Ohki E., Yokoyama H., Ishii H. (2000). Effect of Acute Ethanol Administration on the Intestinal Absorption of Endotoxin in Rats. Alcohol. Clin. Exp. Res..

[87] Bode C., Kugler V., Bode J. (1987). Endotoxemia in patients with alcoholic and non-alcoholic cirrhosis and in subjects with no evidence of chronic liver disease following acute alcohol excess. J. Hepatol..

[88] Mathurin P., Deng Q., Keshavarzian A., Choudhary S., Holmes E., Tsukamoto H. (2000). Exacerbation of Alcoholic Liver Injury by Enteral Endotoxin in Rats. Hepatology.

[89] Enomoto N., Ikejima K., Bradford B., Rivera C., Kono H., Brenner D.A., Thurman R.G. (1998). Alcohol causes both tolerance and sensitization of rat Kupffer cells via mechanisms dependent on endotoxin. Gastroenterology.

[90] Luther J., Garber J.J., Khalili H., Dave M., Bale S.S., Jindal R., Motola D.L., Luther S., Bohr S., Jeoung S.W. (2015). Hepatic Injury in Nonalcoholic Steatohepatitis Contributes to Altered Intestinal Permeability. Cell. Mol. Gastroenterol. Hepatol..

[91] Jennison E., Byrne C.D. (2021). The role of the gut microbiome and diet in the pathogenesis of non-alcoholic fatty liver disease. Clin. Mol. Hepatol..

[92] Gäbele E., Dostert K., Hofmann C., Wiest R., Schölmerich J., Hellerbrand C., Obermeier F. (2011). DSS induced colitis increases portal LPS levels and enhances hepatic inflammation and fibrogenesis in experimental NASH. J. Hepatol..

[93] Henao-Mejia J., Elinav E., Jin C., Hao L., Mehal W.Z., Strowig T., Thaiss C.A., Kau A., Eisenbarth S., Jurczak M. (2012). Inflammasome-mediated dysbiosis regulates progression of NAFLD and obesity. Nat. Cell Biol..

[94] Ludwig J., Viggiano T.R., McGill D.B., Ott B.J. (1980). Nonalcoholic steatohepatitis. Mayo Clinic experiences with a hitherto un-named disease. Mayo Clin. Proc..

[95] Bugianesi E., Gastaldelli A., Vanni E., Gambino R., Cassader M., Baldi S., Ponti V., Pagano G., Ferrannini E., Rizzetto M. (2005). Insulin resistance in non-diabetic patients with non-alcoholic fatty liver disease: Sites and mechanisms. Diabetologia.

[96] Fabbrini E., Sullivan S., Klein S. (2010). Obesity and nonalcoholic fatty liver disease: Biochemical, metabolic, and clinical implications. Hepatology.

[97] Vega G.L., Chandalia M., Szczepaniak L.S., Grundy S.M. (2007). Metabolic correlates of nonalcoholic fatty liver in women and men. Hepatology.

[98] Sanyal A.J., Campbell–Sargent C., Mirshahi F., Rizzo W.B., Contos M.J., Sterling R.K., Luketic V.A., Shiffman M.L., Clore J.N. (2001). Nonalcoholic steatohepatitis: Association of insulin resistance and mitochondrial abnormalities. Gastroenterology.

[99] Abenavoli L., Milic N., Di Renzo L., Preveden T., Medić-Stojanoska M., De Lorenzo A. (2016). Metabolic aspects of adult patients with nonalcoholic fatty liver disease. World J. Gastroenterol..

[100] Almeda-Valdes P., Aguilar-Olivos N., Uribe M., Méndez-Sánchez N. (2015). Common features of the metabolic syndrome and nonalcoholic fatty liver disease. Rev. Recent Clin. Trials.

[101] Buzzetti E., Pinzani M., Tsochatzis E.A. (2016). The multiple-hit pathogenesis of non-alcoholic fatty liver disease (NAFLD). Metabolism.

[102] Eslam M., Sanyal A.J., George J. (2020). International Consensus Panel. MAFLD: A Consensus-Driven Proposed Nomenclature for Metabolic Associated Fatty Liver Disease. Gastroenterology.

[103] El-Serag H.B., Kanwal F., Feng Z., Marrero J.A., Khaderi S., Singal A.G. (2020). Risk Factors for Cirrhosis in Contemporary Hepatology Practices—Findings From the Texas Hepatocellular Carcinoma Consortium Cohort. Gastroenterology.

[104] Bianco C., Casirati E., Malvestiti F., Valenti L. (2021). Genetic predisposition similarities between NASH and ASH: Identification of new therapeutic targets. JHEP Rep..

[105] Tilg H., Moschen A. (2010). Evolution of inflammation in nonalcoholic fatty liver disease: The multiple parallel hits hypothesis. Hepatology.

[106] Bessone F., Razori M.V., Roma M.G. (2019). Molecular pathways of nonalcoholic fatty liver disease development and progression. Cell. Mol. Life Sci..

[107] Masarone M., Rosato V., Dallio M., Gravina A.G., Aglitti A., Loguercio C., Federico A., Persico M. (2018). Role of Oxidative Stress in Pathophysiology of Nonalcoholic Fatty Liver Disease. Oxid. Med. Cell. Longev..

[108] Pan M., Cederbaum A.I., Zhang Y.-L., Ginsberg H.N., Williams K.J., Fisher E.A. (2004). Lipid peroxidation and oxidant stress regulate hepatic apolipoprotein B degradation and VLDL production. J. Clin. Investig..

[109] Weltman M.D., Farrell G.C., Liddle C. (1996). Increased hepatocyte CYP2E1 expression in a rat nutritional model of hepatic steatosis with inflammation. Gastroenterology.

[110] Brunt E.M., Janney C.G., Di Bisceglie A.M., Neuschwander-Tetri B.A., Bacon B.R. (1999). Nonalcoholic Steatohepatitis: A Proposal for Grading and Staging The Histological Lesions. Am. J. Gastroenterol..

[111] Weltman M.D., Farrell G.C., Hall P., Ingelman-Sundberg M., Liddle C. (1998). Hepatic cytochrome P450 2E1 is increased in patients with nonalcoholic steatohepatitis. Hepatology.

[112] Aubert J., Begriche K., Knockaert L., Robin M., Fromenty B. (2011). Increased expression of cytochrome P450 2E1 in nonalcoholic fatty liver disease: Mechanisms and pathophysiological role. Clin. Res. Hepatol. Gastroenterol..

[113] Videla L.A., Rodrigo R., Orellana M., Fernandez V., Tapia G., Quiñones L., Varela N., Contreras J., Lazarte R., Csendes A. (2004). Oxidative stress-related parameters in the liver of non-alcoholic fatty liver disease patients. Clin. Sci..

[114] Zangar R.C., Novak R.F. (1997). Effects of Fatty Acids and Ketone Bodies on Cytochromes P450 2B, 4A, and 2E1 Expression in Primary Cultured Rat Hepatocytes. Arch. Biochem. Biophys..

[115] Woodcroft K.J., Novak R.F. (1997). Insulin effects on CYP2E1, 2B, 3A, and 4A expression in primary cultured rat hepatocytes. Chem. Interact..

[116] Zong H., Armoni M., Harel C., Karnieli E., Pessin J.E. (2012). Cytochrome P-450 CYP2E1 knockout mice are protected against high-fat diet-induced obesity and insulin resistance. Am. J. Physiol. Metab..

[117] Lee H.Y., Lee G.-H., Bhattarai K.R., Park B.-H., Koo S.-H., Kim H.-R., Chae H.J. (2016). Bax Inhibitor-1 regulates hepatic lipid accumulation via ApoB secretion. Sci. Rep..

[118] Kim H.-R., Lee G.-H., Cho E.Y., Chae S.-W., Ahn T., Chae H.-J. (2009). Bax inhibitor 1 regulates ER-stress-induced ROS accumulation through the regulation of cytochrome P450 2E1. J. Cell Sci..

[119] Kim S.-M., Grenert J.P., Patterson C., Correia M.A. (2016). CHIP−/−-Mouse Liver: Adiponectin-AMPK-FOXO-Activation Overrides CYP2E1-Elicited JNK1-Activation, Delaying Onset of NASH: Therapeutic Implications. Sci. Rep..

[120] Leclercq I.A., Farrell G.C., Field J., Bell D.R., Gonzalez F.J., Robertson G.R. (2000). CYP2E1 and CYP4A as microsomal catalysts of lipid peroxides in murine nonalcoholic steatohepatitis. J. Clin. Investig..

[121] Adas F., Berthou F., Salaün J., Dréano Y., Amet Y. (1999). Interspecies variations in fatty acid hydroxylations involving cytochromes P450 2E1 and 4A. Toxicol. Lett..

[122] Adas F., Salaün J., Berthou F., Picart D., Simon B., Amet Y. (1999). Requirement for ω and (ω–1)-hydroxylations of fatty acids by human cytochromes P450 2E1 and 4A11. J. Lipid Res..

[123] Gao H., Cao Y., Xia H., Zhu X., Jin Y. (2020). CYP4A11 is involved in the development of nonalcoholic fatty liver disease via ROS-induced lipid peroxidation and inflammation. Int. J. Mol. Med..

[124] Diesinger T., Buko V., Lautwein A., Dvorsky R., Belonovskaya E., Lukivskaya O., Naruta E., Kirko S., Andreev V., Buckert D. (2020). Drug targeting CYP2E1 for the treatment of early-stage alcoholic steatohepatitis. PLoS ONE.

[125] Diesinger T., Lautwein A., Buko V., Belonovskaya E., Lukivskaya O., Naruta E., Kirko S., Andreev V., Dvorsky R., Buckert D. (2021). ω-Imidazolyl-alkyl derivatives as new preclinical drug candidates for treating non-alcoholic steatohepatitis. Physiol. Rep..

[126] Lambert J.E., Ramos–Roman M.A., Browning J.D., Parks E. (2014). Increased De Novo Lipogenesis Is a Distinct Characteristic of Individuals With Nonalcoholic Fatty Liver Disease. Gastroenterology.

[127] Buyco D.G., Martin J., Jeon S., Hooks R., Lin C., Carr R. (2021). Experimental models of metabolic and alcoholic fatty liver disease. World J. Gastroenterol..

[128] Houten S.M., Wanders R.J.A. (2010). A general introduction to the biochemistry of mitochondrial fatty acid β-oxidation. J. Inherit. Metab. Dis..

[129] Berardo C., Di Pasqua L.G., Cagna M., Richelmi P., Vairetti M., Ferrigno A. (2020). Nonalcoholic Fatty Liver Disease and Non-Alcoholic Steatohepatitis: Current Issues and Future Perspectives in Preclinical and Clinical Research. Int. J. Mol. Sci..

[130] Yoon M. (2009). The role of PPARα in lipid metabolism and obesity: Focusing on the effects of estrogen on PPARα actions. Pharmacol. Res..

[131] Kroetz D.L., Yook P., Costet P., Bianchi P., Pineau T. (1998). Peroxisome Proliferator-activated Receptor α Controls the Hepatic CYP4A Induction Adaptive Response to Starvation and Diabetes. J. Biol. Chem..

[132] Wan Y. (2001). Regulation of peroxisome proliferator activated receptor α-mediated pathways in alcohol fed cytochrome P450 2E1 deficient mice. Hepatol. Res..

[133] Abdelmegeed M.A., Yoo S.-H., Henderson L.E., Gonzalez F.J., Woodcroft K.J., Song B.-J. (2011). PPARα Expression Protects Male Mice from High Fat–Induced Nonalcoholic Fatty Liver. J. Nutr..

[134] Anandatheerthavarada H.K., Addya S., Dwivedi R.S., Biswas G., Mullick J., Avadhani N.G. (1997). Localization of Multiple Forms of Inducible Cytochromes P450 in Rat Liver Mitochondria: Immunological Characteristics and Patterns of Xenobiotic Substrate Metabolism. Arch. Biochem. Biophys..

[135] Robin M.-A., Anandatheerthavarada H.K., Fang J.-K., Cudic M., Otvos L., Avadhani N.G. (2001). Mitochondrial Targeted Cytochrome P450 2E1 (P450 MT5) Contains an Intact N Terminus and Requires Mitochondrial Specific Electron Transfer Proteins for Activity. J. Biol. Chem..

[136] Hartman J.H., Miller G., Meyer J.N. (2017). Toxicological implications of mitochondrial localization of CYP2E1. Toxicol. Res..

[137] Bansal S., Liu C.-P., Sepuri N.B., Anandatheerthavarada H.K., Selvaraj V., Hoek J., Milne G., Guengerich F.P., Avadhani N.G. (2010). Mitochondria-targeted Cytochrome P450 2E1 Induces Oxidative Damage and Augments Alcohol-mediated Oxidative Stress. J. Biol. Chem..

[138] Robin M.-A., Sauvage I., Grandperret T., Descatoire V., Pessayre D., Fromenty B. (2005). Ethanol increases mitochondrial cytochrome P450 2E1 in mouse liver and rat hepatocytes. FEBS Lett..

[139] Li H., Zhou Y., Wang H., Zhang M., Qiu P., Zhang R., Zhao Q., Liu J. (2020). Crosstalk Between Liver Macrophages and Surrounding Cells in Nonalcoholic Steatohepatitis. Front. Immunol..

[140] Mihm S. (2018). Danger-Associated Molecular Patterns (DAMPs): Molecular Triggers for Sterile Inflammation in the Liver. Int. J. Mol. Sci..

[141] Pessayre D., Berson A., Fromenty B., Mansouri A. (2001). Mitochondria in Steatohepatitis. Semin. Liver Dis..

[142] Cao Q., Mak K.M., Lieber C.S. (2005). Cytochrome P4502E1 primes macrophages to increase TNF-α production in response to lipopolysaccharide. Am. J. Physiol. Liver Physiol..

[143] Kim S.Y., Jeong J.-M., Kim S.J., Seo W., Kim M.-H., Choi W.-M., Yoo W., Lee J.-H., Shim Y.-R., Yi H.-S. (2017). Pro-inflammatory hepatic macrophages generate ROS through NADPH oxidase 2 via endocytosis of monomeric TLR4–MD2 complex. Nat. Commun..

[144] Chance B., Sies H., Boveris A. (1979). Hydroperoxide metabolism in mammalian organs. Physiol. Rev..

[145] Ingelman-Sundberg M., Johansson I. (1980). Cytochrome b5 as electron donor to rabbit liver cytochrome P-450LM2 in reconstituted phospholipid vesicles. Biochem. Biophys. Res. Commun..

[146] White R.E. (1991). The involvement of free radicals in the mechanisms of monooxygenases. Pharmacol. Ther..

[147] Linhart K., Bartsch H., Seitz H.K. (2014). The role of reactive oxygen species (ROS) and cytochrome P-450 2E1 in the generation of carcinogenic etheno-DNA adducts. Redox Biol..

[148] Schattenberg J.M., Czaja M.J. (2014). Regulation of the effects of CYP2E1-induced oxidative stress by JNK signaling. Redox Biol..

[149] Liu R.-M., Desai L.P. (2015). Reciprocal regulation of TGF-β and reactive oxygen species: A perverse cycle for fibrosis. Redox Biol..

[150] Miñana J.B., Gómez-Cambronero L., Lloret A., Pallardó F.V., Del Olmo J., Escudero A., Rodrigo J.M., Pellíin A., Viña J.R., Viña J. (2002). Mitochondrial oxidative stress and CD95 ligand: A dual mechanism for hepatocyte apoptosis in chronic alcoholism. Hepatology.

[151] Ganji S.H., Kashyap M., Kamanna V.S. (2015). Niacin inhibits fat accumulation, oxidative stress, and inflammatory cytokine IL-8 in cultured hepatocytes: Impact on non-alcoholic fatty liver disease. Metabolism.

[152] Yin M., Gäbele E., Wheeler M.D., Connor H., Bradford B.U., Dikalova A., Rusyn I., Mason R., Thurman R.G. (2001). Alcohol-induced free radicals in mice: Direct toxicants or signaling molecules?. Hepatology.

[153] Pessayre D., Fromenty B. (2005). NASH: A mitochondrial disease. J. Hepatol..

[154] Loguercio C., De Girolamo V., de Sio I., Tuccillo C., Ascione A., Baldi F., Budillon G., Cimino L., Di Carlo A., Di Marino M.P. (2001). Non-alcoholic fatty liver disease in an area of southern Italy: Main clinical, histological, and pathophysiological aspects. J. Hepatol..

[155] Begriche K., Igoudjil A., Pessayre D., Fromenty B. (2006). Mitochondrial dysfunction in NASH: Causes, consequences and possible means to prevent it. Mitochondrion.

[156] Begriche K., Knockaert L., Massart J., Robin M.-A., Fromenty B. (2009). Mitochondrial dysfunction in nonalcoholic steatohepatitis (NASH): Are there drugs able to improve it?. Drug Discov. Today Dis. Mech..

[157] Abdel-Maboud M., Menshawy A., Menshawy E., Emara A., Alshandidy M., Eid M. (2020). The efficacy of vitamin E in reducing non-alcoholic fatty liver disease: A systematic review, meta-analysis, and meta-regression. Ther. Adv. Gastroenterol..

[158] Amanullah I., Khan Y.H., Anwar I., Gulzar A., Mallhi T.H., Raja A.A. (2019). Effect of vitamin E in non-alcoholic fatty liver disease: A systematic review and meta-analysis of randomised controlled trials. Postgrad. Med. J..

[159] Schürks M., Glynn R.J., Rist P.M., Tzourio C., Kurth T. (2010). Effects of vitamin E on stroke subtypes: Meta-analysis of randomised controlled trials. BMJ.

[160] Wang T., Xu L. (2019). Circulating Vitamin E Levels and Risk of Coronary Artery Disease and Myocardial Infarction: A Mendelian Randomization Study. Nutrients.

[161] Klein E.A., Thompson I.M., Tangen C.M., Crowley J.J., Lucia M.S., Goodman P.J., Minasian L.M., Ford L.G., Parnes H.L., Gaziano J.M. (2011). Vitamin E and the Risk of Prostate Cancer: The selenium and vitamin e cancer prevention trial (SELECT). JAMA.

[162] Wang K., Tan W., Liu X., Deng L., Huang L., Wang X., Gao X. (2021). New insight and potential therapy for NAFLD: CYP2E1 and flavonoids. Biomed. Pharmacother..

[163] Yao H., Qiao Y.-J., Zhao Y.-L., Tao X.-F., Xu L.-N., Yin L.-H., Qi Y., Peng J.-Y. (2016). Herbal medicines and nonalcoholic fatty liver disease. World J. Gastroenterol..

[164] Hurrell T., Kastrinou-Lampou V., Fardellas A., Hendriks D.F.G., Nordling Å., Johansson I., Baze A., Parmentier C., Richert L., Ingelman-Sundberg M. (2020). Human Liver Spheroids as a Model to Study Aetiology and Treatment of Hepatic Fibrosis. Cells.

[165] Soret P.-A., Magusto J., Housset C., Gautheron J. (2020). In Vitro and In Vivo Models of Non-Alcoholic Fatty Liver Disease: A Critical Appraisal. J. Clin. Med..

[166] Kozyra M., Johansson I., Nordling Å., Ullah S., Lauschke V.M., Ingelman-Sundberg M. (2018). Human hepatic 3D spheroids as a model for steatosis and insulin resistance. Sci. Rep..

[167] Vorrink S.U., Ullah S., Schmidt S., Nandania J., Velagapudi V., Beck O., Ingelman-Sundberg M., Lauschke V.M. (2017). Endogenous and xenobiotic metabolic stability of primary human hepatocytes in long-term 3D spheroid cultures revealed by a combination of targeted and untargeted metabolomics. FASEB J..

[168] Prill S., Caddeo A., Baselli G.A., Jamialahmadi O., Dongiovanni P., Rametta R., Kanebratt K.P., Pujia A., Pingitore P., Mancina R.M. (2019). The TM6SF2 E167K genetic variant induces lipid biosynthesis and reduces apolipoprotein B secretion in human hepatic 3D spheroids. Sci. Rep..

